# Solitary pulmonary nodule and the surgeon

**DOI:** 10.7196/AJTCCM.2020.v26i1.053

**Published:** 2020-03-19

**Authors:** K J Dullabh, K Maharaj

**Affiliations:** Department of Cardiothoracic Surgery, Inkosi Albert Luthuli Central Hospital, University of KwaZulu-Natal, Durban, South Africa

**Keywords:** SPN, lobectomy, sub solid nodule, solid nodule

## Abstract

A solitary pulmonary nodule is a single, well-circumscribed radiographic opacity that will be encountered by every thoracic surgeon, and management is dependent on the malignant potential of the nodule. The nodules are usually first encountered on a chest radiograph. Anatomical characteristics on computed tomography can help to better differentiate the malignant potential of the nodule. These characteristics include nodule size, volume change over time, edge morphology, presence of calcification and nodule attenuation. Other adjuncts to evaluate the malignant potential of the nodule include a functional assessment using positron emission tomography. The role of the thoracic surgeon includes both diagnostic and surgical intervention to assist with management of the malignant nodule.

## Background

A solitary pulmonary nodule (SPN) is a common clinical entity in the practice of thoracic surgery, and its management has historically been dependent on factors such as the patient’s age, presence of calcification and changes of nodule size on serial imaging.^[1]^ However, with the advent of improved technology, SPN management has evolved, both in terms of screening and surgical intervention. Most incidental nodules encountered by the surgeon will be benign. With early detection and effective treatment of the malignant nodule, survival may be improved.^[2]^ In countries with a high prevalence of inflammatory lung disease, SPN poses a unique challenge, as the goal is to determine which nodules require intervention or conservative treatment. The goal from a surgical viewpoint is to identify the potentially malignant nodule and reduce unnecessary thoracotomies for benign conditions.

A SPN is defined as a single, well-circumscribed, radiographic opacity ≤30 mm at its widest diameter that is completely surrounded by aerated lung parenchyma, and is not associated with atelectasis, hilar enlargement or pleural effusions.^[3]^ The prevalence of SPN on non-screening chest radiographs (CXR) and computed tomography (CT) varies between 0.09% and 7%, and 8% and 51%, respectively.^[4]^ The differential diagnoses (Table 1) for a SPN span a wide clinical spectrum, from an infectious granuloma to malignant bronchial carcinoma.

## Importance of early detection of the malignant SPN

The goal of the evaluation of a SPN is to identify the malignant nodule so that effective, potentially curative, treatment can be initiated. A malignant SPN can represent early stage lung cancer (LC). Early detection allows the surgeon to control the disease process, by surgically removing it and thereby reducing the development of metastasis. LC can be differentiated into two main groups, non-small-cell lung cancer (NSCLC) and small-cell lung cancer (SCLC). According to the Global Cancer statistics^[5]^ in 2018, LC is the most commonly diagnosed cancer in both sexes (11.6% of all cancers). It is the leading cause of death in males, and the third leading cause of deaths in females.^[5]^ It is estimated that LC accounted for 18.4% of global cancer mortality and ~17% cancer-related deaths in South Africa (SA).^[5]^ For NSCLC, the 5-year survival of a T1a malignancy is 92%, T1b is 83% and T1c is 77%, compared with a T2a nodule, where it is estimated that the 5-year survival drops to 60%.^[6]^ Once diagnosed, appropriate surgical intervention can be undertaken to reduce the recurrence rate of LC to 11%.^[7]^

## General evaluation of a SPN

The general approach for a patient who presents with a SPN centres on the likelihood of malignancy. A thorough history is imperative to identify risk factors for malignancy. According to the American College of Chest Physicians (ACCP), individuals who are at risk of LC can be divided into those who have had exposure to aetiological agents, and those who are susceptible to these agents.^[8]^ Therefore, the history should focus on present or past exposure to tobacco smoking or second-hand smoke, a previous history of malignancy or any family history of malignancy. Other exposure includes heavy metals, asbestos and radon exposure. A thorough clinical examination helps one identify the sequelae of malignancy.

**Fig. 1:**
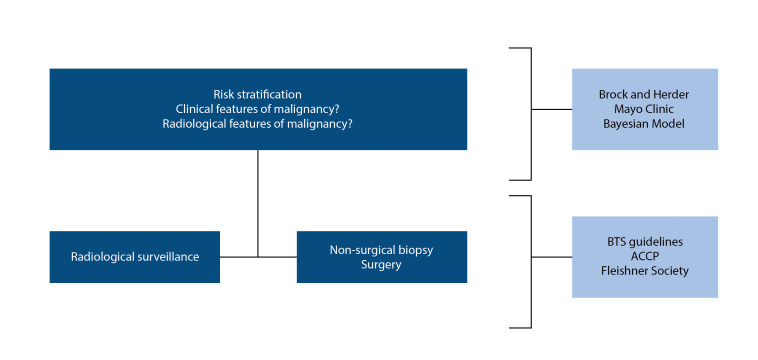
General approach to a solitary pulmonary nodule. BTS = British Thoracic Society ACCP = American College of Chest Physicians

## Role of the basic chest radiograph

The CXR is usually the first investigation undertaken, especially in a resource-limited setting such as SA. On occasion, patients may present with an incidental finding of a SPN when they are undergoing investigations for cardiac pathology, or for routine screening for other medical procedures or even health assessment for work. SPNs have been noted in 0.09% - 0.2% of radiographs.^[9]^ Nodules as small as 5 - 6 mm can be visualised on a CXR.^[10]^ In a resource-limited setting, serial chest radiographs can serve as a an inexpensive method of establishing baseline characteristics of the nodule, and can also be used as a simple screening method to monitor interval changes of the nodule size or number.

## Role of computed tomography

Through the advent of CT and highresolution images through finer CT imaging slices, the detection of nodules has increased. SPNs were noted in up to 50% of thin-slice CT scans. The National Lung Screening Trial (NLST) was a large randomised controlled trial that showed that screening for LC resulted in a 20% reduction in mortality in high-risk groups.^[11]^ However, it had limitations in that it also resulted in a high number of false positives. CT imaging, with its better resolution, can aid further characterisation of the nodule, and owing to its higher sensitivity and specificity compared with CXR, it can provide specific information about size, location and attenuation of the nodule. It can detect both solid and, with newer modalities and thinner image slices, subsolid nodules as well. In surgical practice it is important to assess the nodule in both the mediastinal and lung settings.

**Fig. 2:**
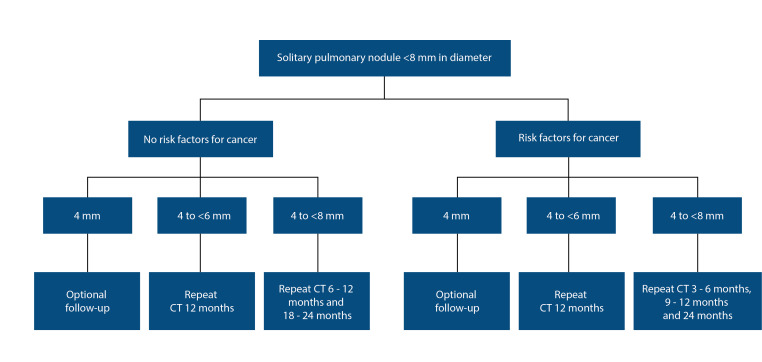
Management of solid pulmonary nodules <8 mm (adapted from American College of Chest Physicians guidelines).^[6]^ CT = computed tomography

## Radiological sign of a SPN

CT allows anatomical definition and characterisation of the nodule. These characteristics are nodule size, nodule volumetric assessment, volume doubling time (VDT), nodule edge morphology, the presence of calcification in the nodule and nodule attenuation, which has been emphasised in recent studies.

### Nodule size

The first evaluation of a nodule on a lowdose CT is of the nodule size. It is well established that the upper limit of a SPN is 30 mm and that nodules larger than this are considered malignant until proven otherwise.^[9]^ However, most guidelines differ regarding the the lower limit of the nodule size that warrants further investigation. The ACCP uses 8 mm, the Fleishner Society uses 6 mm and the British Thoracic Society uses 5 mm as the lower limit for further investigation of a nodule. Data obtained from the NLST showed that >90% of nodules <20 mm in diameter were benign. Further analyses show that nodules that are between 7 and 10 mm have a 1.7% probability of being malignant, nodules that are 11 - 20 mm have 11.9% probability and nodules that are 21 - 30 mm have a 41.3% probability of being malignant.^[12]^ The likelihood of malignancy is directly proportional to the size of the nodule, and is universally included as a a key characteristic in the evaluation of SPNs.

**Fig. 3:**
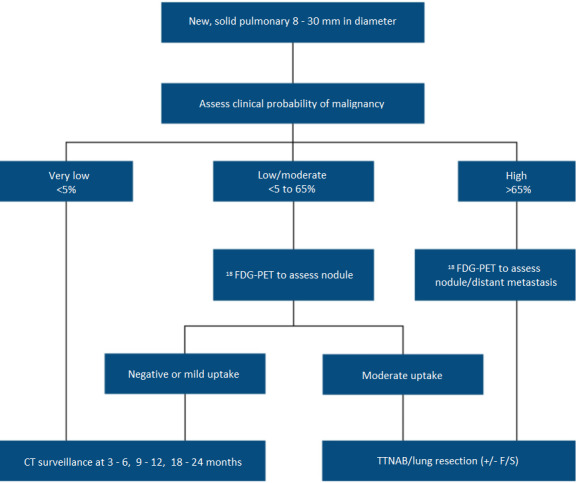
Management of solid pulmonary nodules >8 mm (adapted from American College of Chest Physicians guidelines).^[6]^ ^18^FDG-PET = ^18^F-fluoro-deoxy-D-glucose positron emission tomography CT = computed tomography TTNAB = transthoracic needle aspiration biopsy F/S = frozen section

### Nodule volumetric assessment and VDT

Bronchogenic carcinoma nodules usually grow over a period of time. CT images are often seen in two-dimensional (2D)planes. However, nodules can grow both superiorly and inferiorly, medially and laterally, as well as anteriorly and posteriorly. A volumetric three-dimensional assessment of a nodule has been found to be more accurate than a 2D assessment.^[13,14]^ The NELSON trial used volumetric assessment of nodules. The trial found that nodules <100 mm³ have a low risk for malignancy, whereas nodules that are >300 mm³ have a 16.9% chance of malignancy.^[15]^

VDT is defined as the number of days it takes for a nodule to double in volume.^[16]^ It has an important impact on prognosis in a patient with a nodule. The VDT from bronchogenic carcinoma is rarely <1 month or >1 year, with an average doubling time of 100 days (range 20 - 400).^[17]^ Usually, nodules that double in size in <20 days are indicative of an inflammatory or infective cause, whereas nodules that have a doubling time of more than 400 days are benign.^[18]^ The caveat to this is that some indolent tumours, such as the subsolid adenocarcinoma, may take up to 1 346 days to double in size.^[19]^ The data from the NELSON trial show that a SPN with a VDT <400 days had a 9.7% probability of cancer at 2 years.

### Nodule edge morphology

**Fig. 4:**
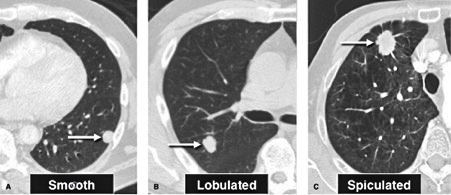
(A) Solitary pulmonary nodule with well-defined regular border usually indicates a benign condition. (B) Lobulation occurs when different parts of the nodule grow at different rates. (C) Spiculation occurs when tumour cells infiltrate the lympathic channels causing septal thickening.

The edge of the nodule can be smooth, lobulated, irregular or speculated (Fig. 4). Smooth borders are generally indicative of benignity; however, up to 21% may be malignant.^[20]^ Lobulated, irregular and spiculated edge morphologies are generally associated with malignancy. Lobulation or an irregular border occurs when different parts of the nodule grow at different rates. Spiculation (also known as corona radiata) consists of fine linear strands extending 4 - 5 mm outward from the nodule, which are interlobular septal thickening and fibrosis due to tumour cells infiltrating lymphatic channels causing obstruction.^[20]^

### Calcification

**Fig. 5:**
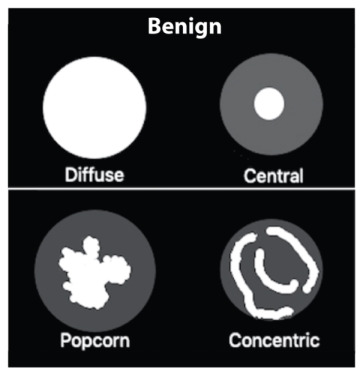
Pattern of benign calcification: diffuse, central, popcorn, concentric.

Calcification occurs when damaged lung parenchyma is replaced by fibrosis. Fibrosis may then heal with calcium deposits. There are different types of calcification patterns that can determine the benignity of a lesion and these are central, diffuse and laminated (Fig. 5). These patterns are typically seen in granulomatous infection such as tuberculosis or histoplasmosis.^[21]^ It is also important to note that diffuse calcification can be seen in metastasis, especially in bone-forming carcinomas such as osteosarcoma. A fourth benign calcification, the so called ‘popcorn’ calcification, is usually a result of chondroid calcification and is typically pathognomonic of a pulmonary harmatoma.

**Fig. 6:**
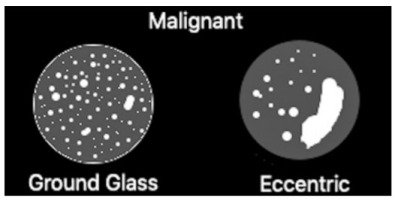
Usual pattern of malignant calcification: ground glass and eccentric

Patterns of calcification in LC are described as eccentric and stippled (Fig. 6). This is caused by the erratic growth of tumour cells, which outgrow the blood supply at different levels and therefore the process of healing with fibrosis and calcification occurs in a disorganised fashion.

### Nodule attenuation

**Fig. 7:**
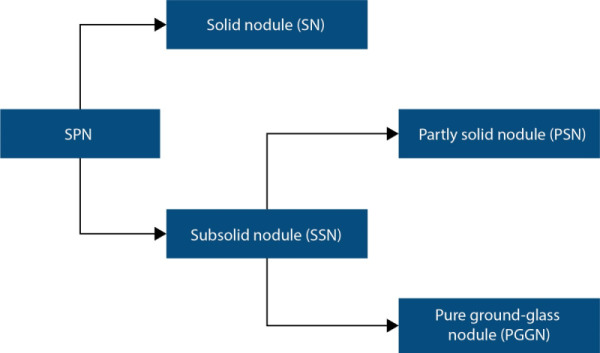
Classification of nodules based on nodule attenuation.^[2]^

In recent years, with the advent of better special resolution of LDCT, there has been a shift of focus on nodule attenuation. Nodules can be divided into solid nodules (SNs) and subsolid nodules (SSNs). Subsolid lesions can be further divided into partly solid nodules (PSNs) and pure ground-glass opacity (pGGO) (Fig. 7).

**Fig. 8:**
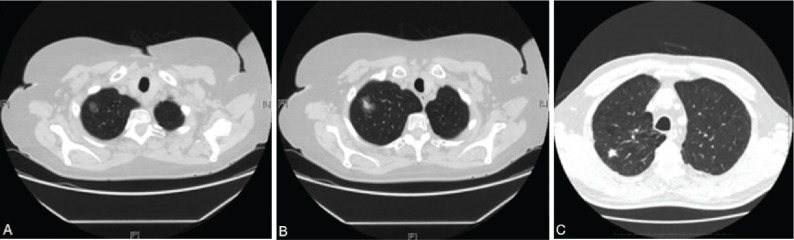
Different attenuations of a nodule seen on a computed tomography scan. (A) A pure ground-glass opacity where the underlying architecture is intact. (B) A subsolid nodule seen where the solid area represents stromal invasion. (C) A solid lesion with complete invasion of the underlying parenchyma

**Fig. 9:**
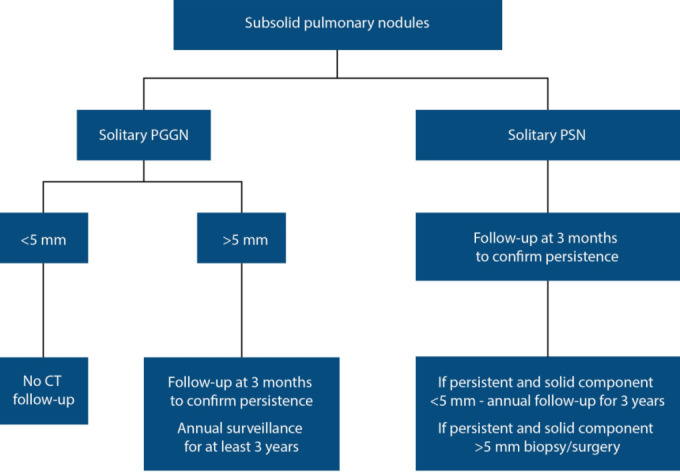
Management of subsolid nodules.^[6]^ PGGN = pure ground-glass nodule PSN = partly solid nodule CT = computed tomography scan

This distinction is important in LC as subsolid lesions may represent early stages of malignancy. However, they do not behave in the same manner as a typical malignancy. It displays a lepidic growth pattern in that the atypical cells proliferate along the alveolar wall. It also has a slow indolent growth rate. The differential for SSNs is just as vast as for SNs. SSNs have the underlying bronchovascular structures visible through them. A pGGO occurs when the opacification on a CT caused by the GGO is greater than that of the background lung tissue, but the underlying vasculature is still preserved (Fig. 8). This opacification can represent atypical adenomatous hyperplasia (AAH) if the diameter is <5 mm. AAH is defined as an irregular-bordered focal proliferation of variable degrees atypia of alveolar epithelial cells but less than that seen in adenocarinoma. It is usually a premalignant condition of pulmonary adenocarcinoma.^[22]^ If the pGGO has a diameter >5 mm, it is called adenocarcinoma in situ (AIS). AIS is defined as a small (<30 mm) adenocarcinoma that does not invade the stroma, vascular or alveolar space or pleura.^[23]^ As part of the lesion becomes solid, the opacity obscures the underlying parenchyma in the lung window settings. This is usually a sign of invasion, and is termed minimally invasive adenocarcinoma (MIA). MIA is defined as a small (<30 mm) solitary adenocarcinoma that also displays a lepidic growth pattern, that has stromal invasion that is <5 mm along its widest dimension and also does not invade the lymphatic, vascular or alveolar space or pleura.^[23]^ Both AIS and MIA represent early-stage adenocarcinoma, and prompt surgical treatment can be offered to the patient. It is therefore imperative to monitor the size of the pGGO and to assess the size of the solid component of the SSN (Fig. 9).

## Role of PET-CT

If there is still diagnostic doubt about a nodule, the next step is to see if it behaves like a malignancy functionally. As an imaging modality, positron emission tomography (PET) has been used extensively in studies in the diagnosis of indeterminate lung lesion. It is superior to CT in screening for extrathoracic disease, and can be used for radiological surveillance of a lung nodule, as well as to assess the response to treatment. In general, malignant cells have a higher rate of glucose metabolism compared with non-malignant cells. ^18^F-fluoro-deoxy-D-glucose (^18^FDG) is a radioactive glucose analogue that behaves like glucose and is also transported into the cell via the glucose transport protein. In neoplastic cells, both ^18^FDG and glucose are transported into the cell; however, ^18^FDG is metabolically trapped and accumulates within the cell after phosphorylation by hexokinase.^[24]^ These so-called ‘hot spots’ would light up on PET. Various studies have reported ranges of 83% - 100% sensitivity and 63% - 90% specificity for the detection of lung cancer with a standard uptake value (SUV) of 2.5.^[25]^ However, the SUV of 2.5 should be used with caution. Inflammatory and infectious conditions also have a tendency to utilise glucose and therefore will have substantial ^18^FDG uptake with a high SUV value. Conversely, indolent or slow-growing malignant tumours, or sub-centimetre tumours may have low levels of glucose uptake, and therefore a low SUV.^[26]^

## Adjuncts to diagnosis

There are a few nonspecific laboratory tests to assist with clinical and treatment decision-making with regard to SPN. These can be divided into haematological and microbiological tests. In patients with an isolated SPN, haematological tests with elevated white cell counts, procalcitonin, erythrocyte sedimentation rate or a combination of tests may point to an inflammatory condition of the lung. Positive hydatid serology can confer a diagnosis of pulmonary hydatid disease. Sputum analysis could include microbiological analysis for inflammatory lung conditions such as tuberculosis or pneumonia, or cytology analysis for malignancy.

## Role of invasive tissue testing 

Once all the relevant investigations have been done, if the clinical suspicion of malignancy is still present, only a histological diagnosis of the lung nodule can confirm if it is a malignancy. Invasive tissue testing can be divided into transthoracic needle aspiration (TTNA) and surgical biopsy, either via videoassisted thoracotomy (VATS) or open lung biopsy (OLB) via a thoracotomy.

### TTNA

TTNA serves as a minimally invasive technique to obtain a tissue diagnosis of peripheral lung nodules. The accuracy of getting a representative sample is enhanced with CT scan guidance, as it can show the location and the anatomic relationship of the nodule and surrounding tissue. The diagnostic accuracy decreases from 90% to 25% when the nodule is <10 mm.^[27]^ However if the nodules are 11 - 20 mm or 21 - 30 mm, the diagnostic accuracy increases to 78.9% and 86.7%, respectively.^[28]^ TTNA is not without complications. These include pneumothorax with an incidence of 10 - 40%, and haemorrhage with the incidence of 26 - 33% in various literature reports.^[29]^ These complications are relatively easy to manage. TTNA can be used to obtain a tissue diagnosis in those patients who are unfit for surgery or for patients refusing surgery. It can also be used to aid the physician or surgeon in terms of the management of the malignant nodule.

### Surgery

Surgery can serve as both a method of diagnosis and treatment. Surgical biopsy is usually reserved for when TTNA fails to give a diagnosis, or when the probability of malignancy is high. In most centres in SA, if the clinical probability of malignancy is high and the patient is a suitable candidate for surgical intervention, thoracic surgeons would undertake a surgical biopsy of the nodule. A surgical biopsy has the advantage both for diagnosis and treatment of a malignant lung nodule. Surgical options include VATS or OLB. Most surgeons opt for the VATS approach as this is minimally invasive and has superior postoperative results to open surgery.^[30]^ If there is diagnostic uncertainty, samples that are taken intraoperatively are sent for frozen section analysis. Lobectomy and lymph node dissection is the treatment of choice for the malignant nodule. Sublobar resections are divided into wedge resections and segmentectomies. These types of resections are limited for diagnostic purposes and are limited to patients with poor pulmonary reserve. Sublobar resections can be performed safely in T1a lesions.^[31]^

## Conclusion

The management of a SPN is not a dichotomous choice between two options, but instead represents a more complicated set of clinical decisions of either discharge, surveillance, functional imaging, biopsy or treatment (either surgical or nonsurgical). It is a clinical entity that all thoracic surgeons will face in their careers. The surgical goal is to find a balance between management of benign and malignant nodules. There are several clues to identify the malignant nodule. These include change in volume and diameter over time, eccentric and GGO calcifications, nodule attenuation and ^18^FDG avidity on PET. In SA we have a high prevelance of inflammatory lung conditions that could mimic the malignant nodule. With early detection of the malignant nodule, appropriate surgical management based on guideline algorithms can be instituted as this could reduce the patient’s morbidity and mortality, especially in the setting of a malignant nodule.

## Figures and Tables

**Table 1:** Differential diagnoses for a solitary pulmonary nodule

Malignant	Benign soft tissue	Infectious	Non-infectious	Congenital
Non-small-cell lung cancer	Hamartoma	Tuberculosis	Rheumatoid	Bronchogenic cyst
Single metastasis	Lipoma	Histoplasmosis	Wegner granulomatosis	
Carcinoid	Fibroma	Coccidioidomycosis	Sarcoidosis	
Small-cell lung cancer		Aspergilloma		
		Ecchinococcal cyst
